# Harpagoside Protects Against Doxorubicin-Induced Cardiotoxicity *via* P53-Parkin-Mediated Mitophagy

**DOI:** 10.3389/fcell.2022.813370

**Published:** 2022-02-10

**Authors:** Weili Li, Xiaoping Wang, Tianhua Liu, Qian Zhang, Jing Cao, Yanyan Jiang, Qianbin Sun, Chun Li, Wei Wang, Yong Wang

**Affiliations:** ^1^ School of Life Science, Beijing University of Chinese Medicine, Beijing, China; ^2^ Modern Research Center for Traditional Chinese Medicine, Beijing University of Chinese Medicine, Beijing, China; ^3^ Beijing Key Laboratory of TCM Syndrome and Formula, Beijing, China; ^4^ Key Laboratory of TCM Syndrome and Formula, Ministry of Education, Beijing University of Chinese Medicine, Beijing, China

**Keywords:** p53, parkin, mitophagy, mitochondria, doxorubicin, cardiotoxicity, harpagoside

## Abstract

Doxorubicin (DOX) is one of the most effective chemotherapeutic agents. However, its clinical use is limited due to the severe risk of cardiotoxicity. One of the hallmarks of doxorubicin-induced cardiotoxicity (DICT) is the cascade of mitophagy deficiency-mitochondrial oxidative injury-apoptosis, while so far, there is no preventive strategy for alleviating DICT by targeting this molecular mechanism. Excitedly, based on our previous drug screen in DICT zebrafish model, harpagoside (HAR) showed dramatic anti-DICT efficacy superior to dexrazoxane (DXZ) only cardioprotectant approved by FDA. Therefore, its pharmacological effects and molecular mechanism on DICT mouse and rat cardiomyocytes were further discussed. *In vivo*, HAR significantly improved cardiac function and myocardial structural lesions with concomitant of diminished mitochondrial oxidative damage and recovered mitophagy flux. In parallel, HAR protected mitophagy and mitochondria homeostasis, and repressed apoptosis *in vitro*. Intriguingly, both nutlin-3 (agonist of p53) and Parkin siRNA reversed these protective effects of HAR. Additional data, including fluorescence colocalization of Parkin and MitoTracker and mt-Keima for the detection of mitophagy flux and coimmunoprecipitation of p53 and Parkin, showed that HAR promoted Parkin translocation to mitochondria and substantially restored Parkin-mediated mitophagy by inhibiting the binding of p53 and Parkin. Importantly, the results of the cell viability demonstrated that cardioprotective effect of HAR did not interfere with anticancer effect of DOX on MCF-7 and HepG2 cells. Our research documented p53-Parkin-mediated cascade of mitophagy deficiency-mitochondrial dyshomeostasis-apoptosis as a pathogenic mechanism and druggable pathway and HAR as a cardioprotection on DICT by acting on novel interaction between p53 and Parkin.

**GRAPHICAL ABSTRACT:**
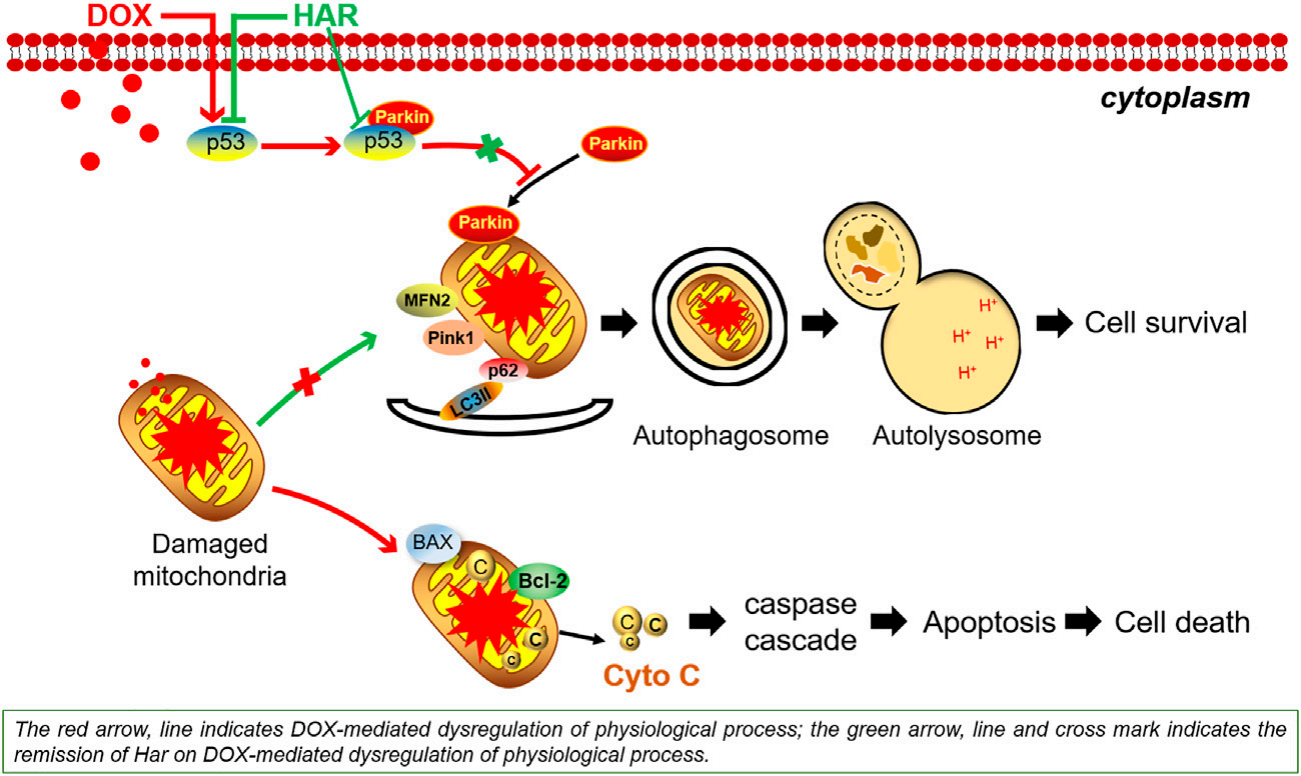


## Introduction

Anthracycline quinone doxorubicin is a potent and broad-spectrum chemotherapeutic agent isolated from *Streptomyces peucetius* in 1967 and has been widely used for the treatment of numerous solid tumours and leukaemias (Blum and Carter, 1974). Despite the effectiveness of the drug, the clinical application of DOX is compromised by dose-dependent and cumulative cardiotoxicity, manifested as cardiomyocyte damage, apoptosis, necrotic cell death and subsequent life-threatening left ventricular dysfunction, cardiomyopathy and/or heart failure (Carvalho et al., 2014; Cardinale et al., 2015). Dexrazoxane is the only cardioprotectant approved by the US Food and Drug Administration for combining with DOX in cancer therapy (Kane et al., 2008). However, dexrazoxane was reported to induce secondary malignancies and aggravate myelosuppression (Shaikh et al., 2015; Tahover et al., 2017). Therefore, exploration of mechanistic processes of pathological occurrence and development of DICT is urgently needed, and new drugs combined with DOX to minimize DICT should be identified (Octavia et al., 2012; Zhang et al., 2012).

The majority of studies on DICT suggested that ROS-mediated mitochondrial impairment events are major aetiological aspect of DOX cardiomyopathy (Govender et al., 2014; Angsutararux et al., 2015; Rochette et al., 2015). Therefore, mitophagy, a mitochondrial quality control mechanism that selectively removes damaged and unwanted mitochondria to maintain a healthy and functional mitochondrial population, has become one of the most promising therapeutic targets for the treatment of DICT (Catanzaro et al., 2019; Wang et al., 2019; Xiao et al., 2020). To date, PTEN-inducible putative kinase 1 (PINK1)/Parkinson juvenile disease protein 2 (Parkin)-mediated mitophagy is the most established mechanism of this process. When mitochondria are depolarized or damaged, Pink1 accumulates at mitochondrial outer membrane and phosphorylates mitofusin2 (Mfn2) as Parkin’s receptor which subsequently can recruit Parkin. Parkin subsequently ubiquitinates multiple proteins, including sequestosome 1 (p62) recognized as ubiquitin- and microtubule-associated protein 1 light chain 3 (LC3)-binding adaptor protein, and this modification is followed by mitophagy (Saito and Sadoshima, 2015; Moyzis and Gustafsson, 2019). A number of studies reported that DOX impedes Parkin-mediated mitophagy, resulting in the accumulation of damaged mitochondria and ultimately decline in cardiac function (Hoshino et al., 2013; Bartlett et al., 2017; Koleini and Kardami, 2017).

Moreover, tumour protein p53 (p53) is a tumour suppressor, which was identified as a multifunctional protein involved in apoptosis and autophagy in various pathologies in recent studies. The p53 gene is lost, mutated or inactivated in most human tumours (Levine et al., 1994), thus p53 has become an ideal target for the treatment of side effects of chemotherapeutic drugs without affecting anticancer ability of these drugs (Ferber, 1999). Intriguingly, DOX can lead to the activation of p53 in the heart (Yoshida et al., 2009; Chang et al., 2011; Velez et al., 2011), which may disturb the clearance of damaged mitochondria *via* an inhibitory interaction with Parkin, and subsequently blocks Parkin translocation to the mitochondria and subsequent mitophagy (Hoshino et al., 2013), finally leading to DICT. Therefore, inhibition of mitophagy mediated by p53-Parkin is considered the main pathogenic alteration in DICT, providing important hints for the development of anti-DICT drugs.

HAR is an monocase of *Scrophularia ningpoensis* that has a variety of pharmacological effects, including anti-inflammatory, neuroprotective and antioxidant effects, in neurological diseases of the brain and osteoarthritis (Sun et al., 2012; Chung et al., 2019; Menghini et al., 2019). However, HAR effect on heart disease has not been reported previously. Our team discovered that HAR has an impressive protective effect on DOX-induced cardiac injury in zebrafish mediated by an unknown mechanism. Therefore, we investigated whether HAR alleviates DOX-induced cardiac injury by regulating the p53-Parkin signalling pathway to restore the mitophagy flux, maintain mitochondrial health and ultimately promote cell survival.

## Methods

### Animals and Pharmacological Treatments

The DICT zebrafish model was established according to the methods described in previous study (Wang et al., 2021). Briefly, at 1 day postfertilization (dpf), zebrafish were treated with 100 μM DOX accompanied with or without 25 μM HAR or 259.89 μM DXZ, and phenotypic changes, including fraction shortening (FS), the blood speed of the tail vein and survival rate were respectively assessed at 3 dpf.

C57BL/6 mice (20 g ± 2 g) were purchased from Beijing SPF Biotechnology (Beijing, China). All experimental procedures and animal care were conducted in accordance with the Guide for the Institutional Animal Care and Use Committee and approved by Beijing University of Chinese Medicine Animal Care Committee. The animals were maintained in a 12:12-h light-dark cycle temperature-controlled environment (22°C) with free access to standard laboratory chow and tap water. A DICT mouse model was generated as described previously (Wang et al., 2020). The mice were randomly assigned to 4 groups of 10 mice each based on the following treatment regimens: mice in the model group, HAR group and enalapril (ENA) group were injected into the tail vein with DOX (5 mg/kg) once weekly for 4 weeks, and mice in the control group were treated with saline solution (0.9% NaCl). HAR (42 mg/kg) and ENA (15 mg/kg) were administered daily orally for 4 weeks 1 week after the last injection of DOX or saline solution, respectively. The control and model groups received the same volume of ultrapure water. The enalapril has been reported to have markedly cardioprotective effects against DICT (Hullin et al., 2018); thus, we used ENA as a positive control in mice experiments.

### Cell Culture and Pharmacological Treatments

H9C2, MCF-7 and HepG2 cells were grown in DMEM supplemented with 10% FBS at 37°C in a humidified atmosphere containing 5% CO_2_. DOX concentrations >2 µM do not reflect a clinically relevant context (Li et al., 2016); hence, we previously used various concentrations of DOX no more than 2 μM; the results confirmed the use of 1 μM DOX as a working concentration. Before co-treating with 1 µM DOX in the presence or absence of HAR (1–500 µM) for 24 h, the H9C2, MCF-7 and HepG2 cells were pretreated with a corresponding concentration of HAR for 24 h. Subsequently, cell viability and other detection were performed. Moreover, H9C2 cells were also treated with 20 μM Pifithrin-α (PFT-α) or 20 μM nutlin-3. PFT-α and nutlin-3 are an inhibitor and an agonist of p53, respectively.

### Echocardiographic Assessment of Cardiac Functions and Histological Examination

The processes were carried out as described previously (Wang et al., 2020).

### RNA Interference

Transfection of Parkin siRNA was performed using transfection reagents and medium according to the manufacturer’s instructions (Hanbio Technology Co., Ltd. Shanghai, China). Cells were transfected with 50 nM Parkin siRNA or negative control siRNA using RNAFit, and the medium was replaced after 24 h incubation with fresh medium, followed by drug treatment. Transfection efficiency was determined by immunoblot.

### Mt-Keima Adenovirus Transfection

The transfection of mt-Keima adenovirus was performed according to the manufacturer’s instructions (Hanbio Technology Co., Ltd. Shanghai, China). H9C2 cells grown on confocal dishes were transfected with mt-Keima adenovirus at multiplicity of infection (MOI) of 50 for 6 h at 37°C. The medium was then discarded and replaced with fresh medium containing the drugs. The cells were observed under confocal microscope. Mt-Keima is a pH-sensitive fluorescent protein, whose excitation spectrum shifts from 440 to 586 nm when mitochondria are delivered to acidic lysosomes, appearing as shift from green to red colour. Mitophagy flux was monitored by evaluating the number of green and red puncta in each cell.

### Co-Immunoprecipitation (Co-IP) Assay

Co-IP assays were performed according to manufacturer’s instructions of immunoprecipitation kit (Cell Signaling Technology, United States). Cytoplasmic extract was incubated with anti-p53 antibody or control IgG1 antibody overnight at 4°C. Protein G magnetic beads were then added to the samples and incubated for 30 min at 4°C. Immunoprecipitates were collected by magnetic separation and washed 5 times. Finally, SDS buffer solution was added to the precipitates, and the samples were heated for 5 min at 98°C for subsequent immunoblot analysis.

### Immunoblot Analysis

The processes were carried out as described previously (Wang et al., 2020). The antibodies used in the study are shown in Table 1.

**TABLE 1:** The antibodies used in this paper.

Antibodies	Companies
p53	10442-1-AP; Proteintech; United States
Parkin	14060-1-AP; Proteintech; United States
Pink1	23274-1-AP; Proteintech; United States
p62	CST88588; Cell Signaling Technology; United States
LC3	14600-1-AP; Proteintech; United States
Bax	50599-2-Ig; Proteintech; United States
Bcl-2	26593-1-AP; Proteintech; United States
Caspase-3	19677-1-AP; Proteintech; United States
Caspase-7	27155-1-AP; Proteintech; United States
Caspase-9	10380-1-AP; Proteintech; United States
GAPDH	CST14C10; Cell Signaling Technology; United States
Mouse IgG1 isotype control	CST5415; Cell Signaling Technology; United States
TOM20	CST42406; Cell Signaling Technology; United States
8-OHdG	Sc-393871; Santa Cruz Biotechnology; United States
VDAC1	CST4866; Cell Signaling Technology; United States

### Statistical Analysis

Statistical analysis was performed using GraphPad software 6. The results are expressed as the mean ± s.d. Statistical significance was evaluated using two-tailed unpaired Student’s t-test. Alternatively, comparisons involving more than two groups were performed by one-way analysis of variance (ANOVA) with Bonferroni post hoc tests for multiple groups to assess the differences. Statistical significance was considered at a *p*-value of <0.05.

## Results

### HAR Relieved DOX-Induced Structural and Functional Cardiac Lesions in DICT Zebrafish and Mouse Model

**FIGURE 1:**
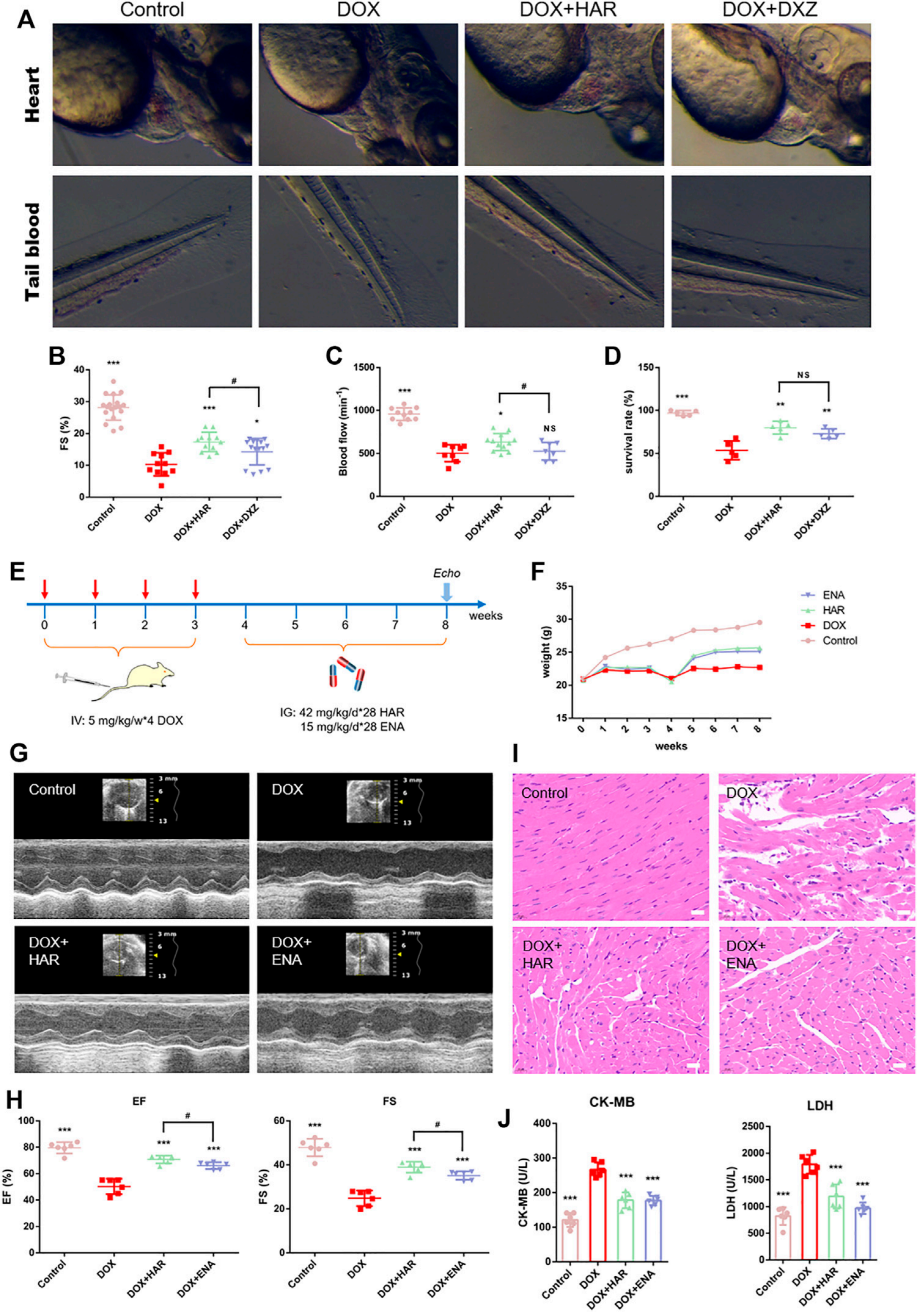
Protective effects of HAR on DICT zebrafish and mice. **(A)** Representative images of zebrafish heart and tail blood flow captured by high-speed camera. **(B,C)** Quantitative analysis of fractional shortening (FS) value of zebrafish hearts and blood flow of zebrafish tail. **(D)** Assessment of survival rate of zebrafish under different drug treatment. **(E)** A schematic diagram of animal model generation and drug administration. **(F)** Body weight changes in various groups (*n* = 10). **(G)** Representative images of echocardiograms. **(H)** Left ventricular ejection fraction and fractional shortening (*n* = 6). **(I)** Representative images of morphological and structural changes in the myocardium detected by H&E staining. Scale bar: 20 μm. **(J)** The content of serum CK-MB and LDH (*n* = 6). ***p < 0.001 vs. DOX group. #*p* < 0.05 vs. (DOX + HAR) group. The data are shown as the mean ± s.d.

We have previously established the DICT zebrafish model and utilized DXZ, a drug approved by FDA for clinical treatment of DICT, as a positive control, to screen TCM monomers with better efficacy than DXZ. Among them, HAR showed a strong anti-DICT effect. As shown and quantified in Figures 1A–D, in DOX group, the fish exhibited extensive pericardial edema (Figure 1A), reduced FS value resulting in decreased blood flow and increased blood accumulation in tail (Figures 1A–C) and ultimately decreased survival rate (Figure 1D). While, HAR alleviated the above harmful changes caused by DOX and was superior to DXZ in protecting FS value of heart and blood flow in tail.

We further evaluated the cardioprotective effect of HAR in DICT mouse model. The design of animal experiments, including generation of DICT model and subsequent pharmacological treatment, is shown in Figure 1E. The body weight of mice was recorded throughout the animal experiment (Figure 1F). DOX resulted in a standstill in weight of mice compared to the sustainedly raised weight in control group, while continued growth in weight of mice were achieved after HAR or ENA administration. The impact of HAR and DOX on the cardiac function of mice were evaluated by echocardiography detection of left ventricular internal diameter at end-diastole (LVID; d), left ventricular internal diameter at end-systole (LVID; s), left ventricular anterior wall at end-diastole (LVAW; d), left ventricular anterior wall at end-systole (LVAW; s), left ventricular posterior wall at end-diastole (LVPW; d) and left ventricular posterior wall at end-systole (LVPW; s). As shown in figure S1, DOX caused increased LVID; d and LVID; s, thinning LVAW; s, LVAW; d, and LVPW; s. HAR and ENA significantly reduced LVID; d and LVID; s, and thickened LVAW; s, and LVPW; s compared to DOX group, while had no effect on LVAW; d and LVPW; d. A significant decrease in the ejection fraction (EF) and fraction shortening (FS) values was observed in the model group compared with those in the control group, and HAR and ENA treatment improved the EF and FS values, and even the EF, FS and LVAW; s values in HAR group were higher than these in ENA group (Figures 1G,H and Figure S1). Consistent with the results of echocardiography, H&E staining in the model group showed myofibrillar loss, disordered arrangement, enlarged intercellular structures and plasma-dissolved cardiomyocytes. However, the pathological changes were partially reversed by HAR and ENA treatment (Figure 1I). Functionally, the levels of serum LDH and CK-MB in the model group were increased compared with those in the control group, and HAR and ENA treatment partially reversed these changes (Figure 1J).

### HAR Repressed DOX-Induced Apoptosis and Mitochondrial Oxidative Stress Damage in DICT Mice

**FIGURE 2:**
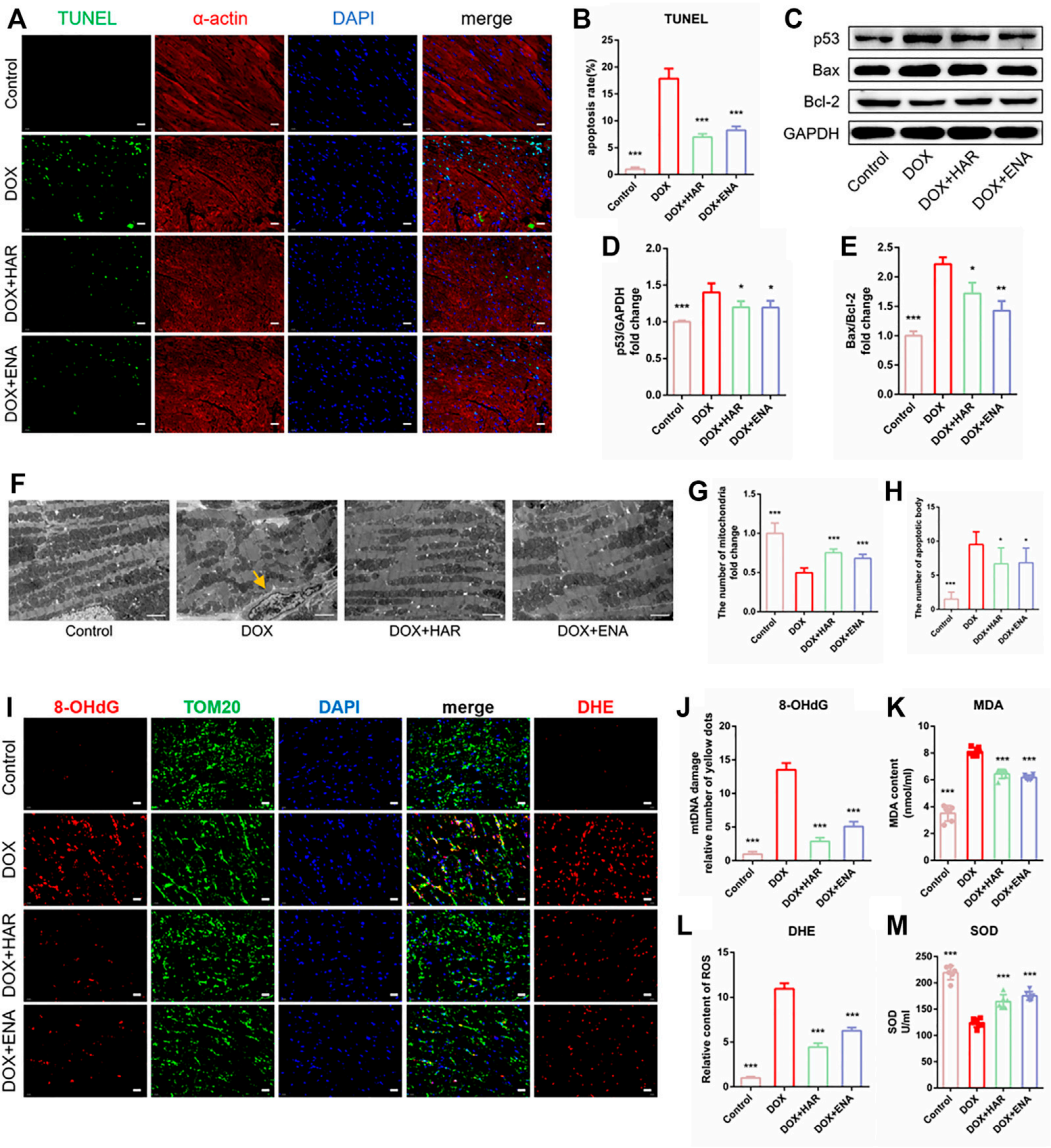
HAR inhibited myocardial apoptosis and alleviated mitochondrial injury in DICT mice. **(A,B)** Representative images and quantitative analysis of apoptosis by TUNEL in mouse cardiac tissues (*n* = 6). Scale bar: 20 μm. **(C–E)**. Representative immunoblots and quantitative analysis of the expression of apoptosis-related proteins mediated by p53 shown as relative protein expression after normalization to GAPDH (*n* = 3–6). **(F,G,H)** Representative electron micrographs displaying mitochondrial arrangement and apoptosis bodies pointed by yellow arrow, and the quantification of mitochondria and apoptosis bodies in mouse hearts. Scale bar: 2.5 μm. The number of mitochondria and apoptosis bodies were quantified blindly using 10 images from 6 samples. **(I,J,K)** Representative images and quantitative analysis of ROS (DHE staining) and damaged mtDNA in mouse cardiac tissues (*n* = 6). Scale bar: 20 μm. Colocalization of 8-OhdG (DNA damage marker) and TOM20 (mitochondrial locator) was used to assess mtDNA damage. **(L, M) **The contents of serum MDA and SOD (*n* = 6). *p < 0.05, **p < 0.01, ***p < 0.001 vs. DOX group. The data are shown as the mean ± s.d.

To detect the effect of HAR on DOX-induced apoptosis, we determined the apoptotic rate of cardiomyocytes by performing TUNEL assay. In addition, α-actin and DAPI were used to label cardiomyocytes and the nuclei, respectively. The results indicated significant apoptosis in the model group (Figures 2A,B), which was in agreement with immunoblot results, showing an increase in the expression of p53 and the ratio of Bcl-2-associated X protein (Bax) to B cell leukaemia/lymphoma 2 protein (Bcl-2) in the model group compared with those in control group, whereas HAR and ENA partially reversed these changes (Figures 2C–E). The results of TEM showed that the number ofapoptotic bodies pointed by yellow arrow increased while mitochondria was decreased, and mitochondria were loosely arranged in the model group, and these effects were suppressed by HAR and ENA (Figures 2F–H). The results of DHE staining suggested that DOX induced a surge in ROS in the cardiac tissue, which was consistent with increased levels of MDA and reduced levels of SOD in the serum in model group. Conversely, HAR and ENA reduced the generation of ROS in the cardiac tissue and MDA in the serum and increased the generation of SOD in the serum (Figures 2I,K–M). 8-Hydroxy-2 deoxyguanosine (8-OHdG), a biomarker of DNA oxidative damage, and translocase of the outer membrane 20 (TOM20), a mitochondrial marker protein, were used to assess the damage of mitochondrial DNA (mtDNA). An increase in mtDNA damage was represented in model group compared with that in control group, whereas mtDNA damage was decreased in HAR and ENA groups (Figures 2I,J).

### HAR Enhanced Parkin-Mediated Mitophagy in DICT Mouse Model

**FIGURE 3:**
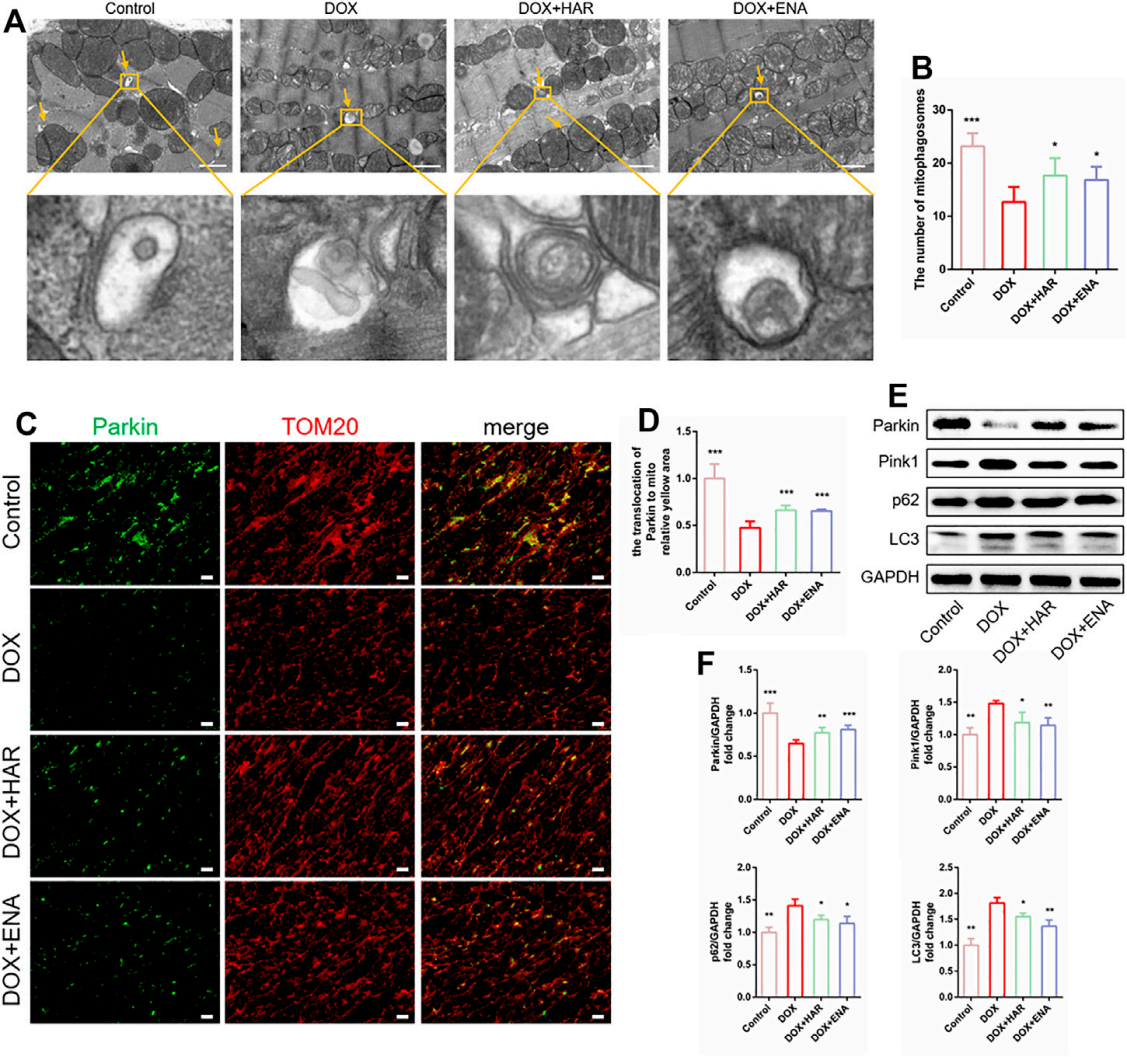
HAR enhanced Parkin-mediated mitophagy in DICT mice. **(A,B)** Representative electron micrographs of the heart and the quantification of mitophagosomes. Scale bar: 1 μm. Magnified image represents an mitophagosomes. The number of mitophagosomes was quantified blindly using 10 images from 6 samples. **(C,D)** Representative images of colocalization of Parkin and TOM20. Scale bar: 20 μm. **(E,F)** Expression levels of p53-dependent mitophagy-associated proteins quantified and shown as relative protein expression levels after normalization to GAPDH. *p < 0.05, **p < 0.01, ***p < 0.001 vs. DOX group (*n* = 3–6). The data are shown as the mean ± s.d.

Mitophagy is a quality control mechanism that maintains mitochondrial homeostasis and plays a prominent role in DICT. TEM can be used to demonstrate the presence of mitochondria within autophagosomes (referred to as mitophagosomes during mitophagy) (Klionsky et al., 2021). TEM results showed that the number of mitophagosomes was lower in the DOX group compared with that in the control group and higher in the HAR and ENA groups than that in the DOX group (Figures 3A,B). Additionally, as shown in Figures 3C,D, colocalization of Parkin and TOM20 was apparently decreased in the DOX group compared with that in the control group and increased in the HAR and ENA groups compared with that in the DOX group. In agreement with these results, mitophagy-related proteins Pink1, p62 and LC3 were significantly increased in the DOX group compared with those in the control group, and this upregulation was partly reversed by HAR and ENA treatment. Instead, DOX intervention reduced Parkin protein expression, and this reduction was reversed by HAR and ENA treatment (Figures 3E,F). These data suggested a hypothesis that inhibition of Parkin by DOX interrupts the formation of mitophagosomes, resulting in the accumulation of Pink1, LC3 and p62, and HAR and ENA upregulate the protein level of Parkin by means of the transcription or translation of Parkin to expedite the formation of mitophagosomes and fully use Pink1, LC3 and p62.

### HAR Mitigated DOX-Induced Apoptosis and Mitochondrial Oxidative Stress Damage by Suppressing p53 *In Vitro*

**FIGURE 4:**
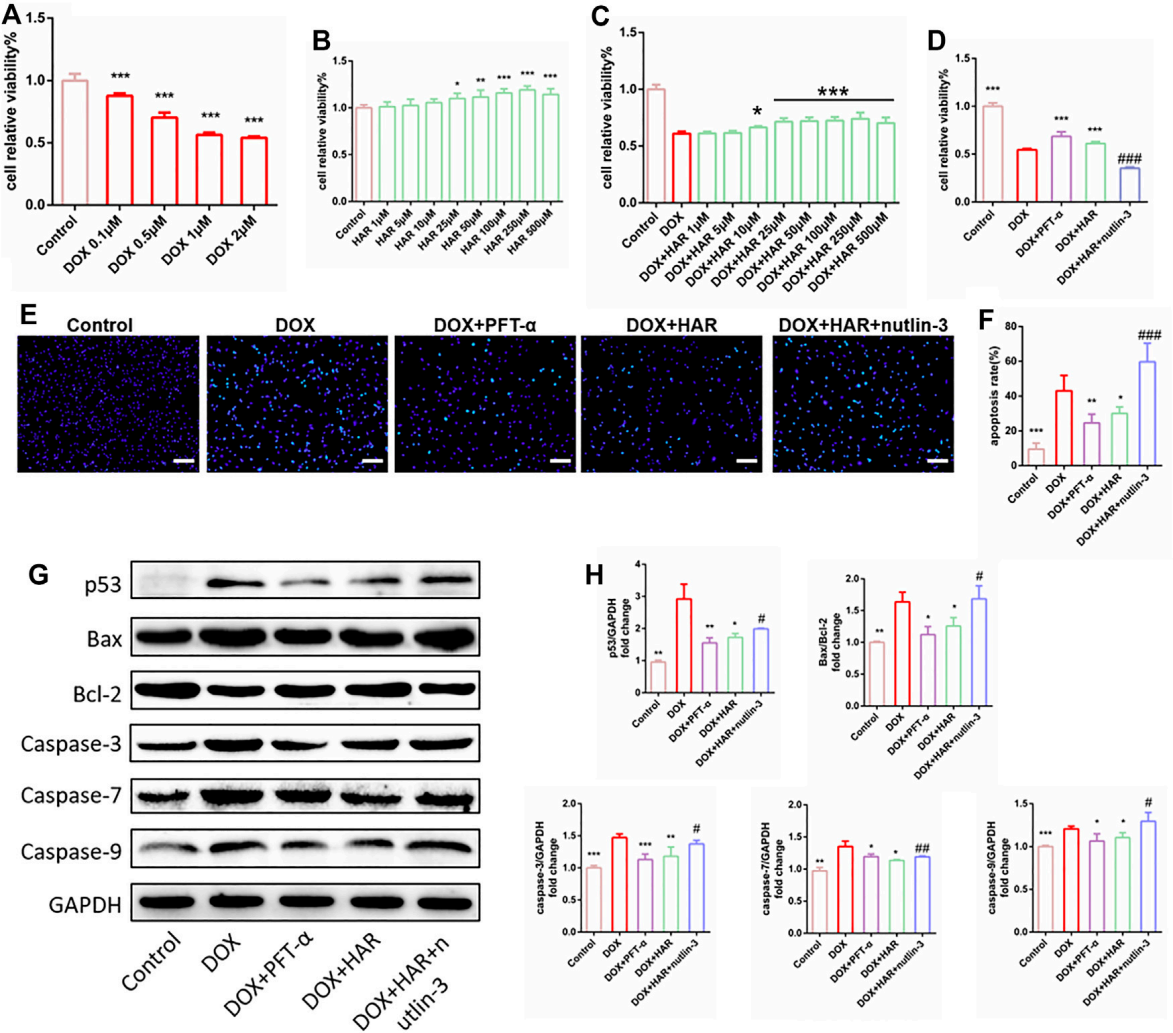
HAR enhanced cell proliferation and inhibited apoptosis by suppressing p53 *in vitro*. **(A)** DOX decreased cell viability in dose-dependent manner, as shown by CCK-8 assay (*n* = 6). **(B,C)** Effect of HAR alone and HAR combined with DOX on cell proliferation (*n* = 6). **(D)** Effects of HAR (pretreatment for 24 h), the p53 inhibitor PFT-α (20 μM) and the p53 activator nutlin-3 (20 μM) on viability of H9C2 cells in the presence of DOX (*n* = 6). **(E,F)** Representative images and quantitative analysis of apoptosis measured by Hoechst staining. Scale bar: 100 μm. Hoechst-positive cells were quantified blindly using 5 images from various fields of view (*n* = 5). **(G,H)** Representative immunoblots and quantitative analysis of the expression of p53-dependent mitochondria-derived apoptosis-related proteins. The expression levels were normalized to GAPDH (*n* = 3–6). *p < 0.05, **p < 0.01, ***p < 0.001 vs. DOX group (*n* = 3–6). ^#^ *p* < 0.05, ^###^ *p* < 0.001 vs. (DOX + HAR) group. The data are shown as the mean ± s.d.

The viability of H9C2 cells was reduced by 40–50% in the presence of 1 μM DOX (Figure 4A). Therefore, 1 μM DOX was used to further evaluate the protective effects of HAR, the p53 inhibitor PFT-α and the p53 activator nutlin-3. HAR among 25–500 μM significantly improved cell viability and proliferation compared to control group (Figure 4B), and HAR among 10–500 μM significantly improved cell viability and proliferation compared to DOX group (Figure 4C). Subsequently, we determined 25 μM HAR for subsequent experiments. As shown in Figure 4D, HAR and PFT-α significantly improve cell viability and proliferation whereas nutlin-3 completely abolished the protective effect of HAR. The results of Hoechst 33,342 staining indicated that HAR and PFT-α reduced apoptosis induced by DOX, and nutlin-3 completely reversed the effect of HAR in agreement with the results of cell viability assays (Figures 4E,F). Furthermore, the expression of apoptosis-associated proteins, including p53, Bax/Bcl-2 and caspases-3/7/9 were increased in DOX group compared to those in control group. These changes were relieved by HAR and PFT-α treatment, and nutlin-3 treatment considerably abrogated the effect of HAR (Figures 4G,H).

**FIGURE 5:**
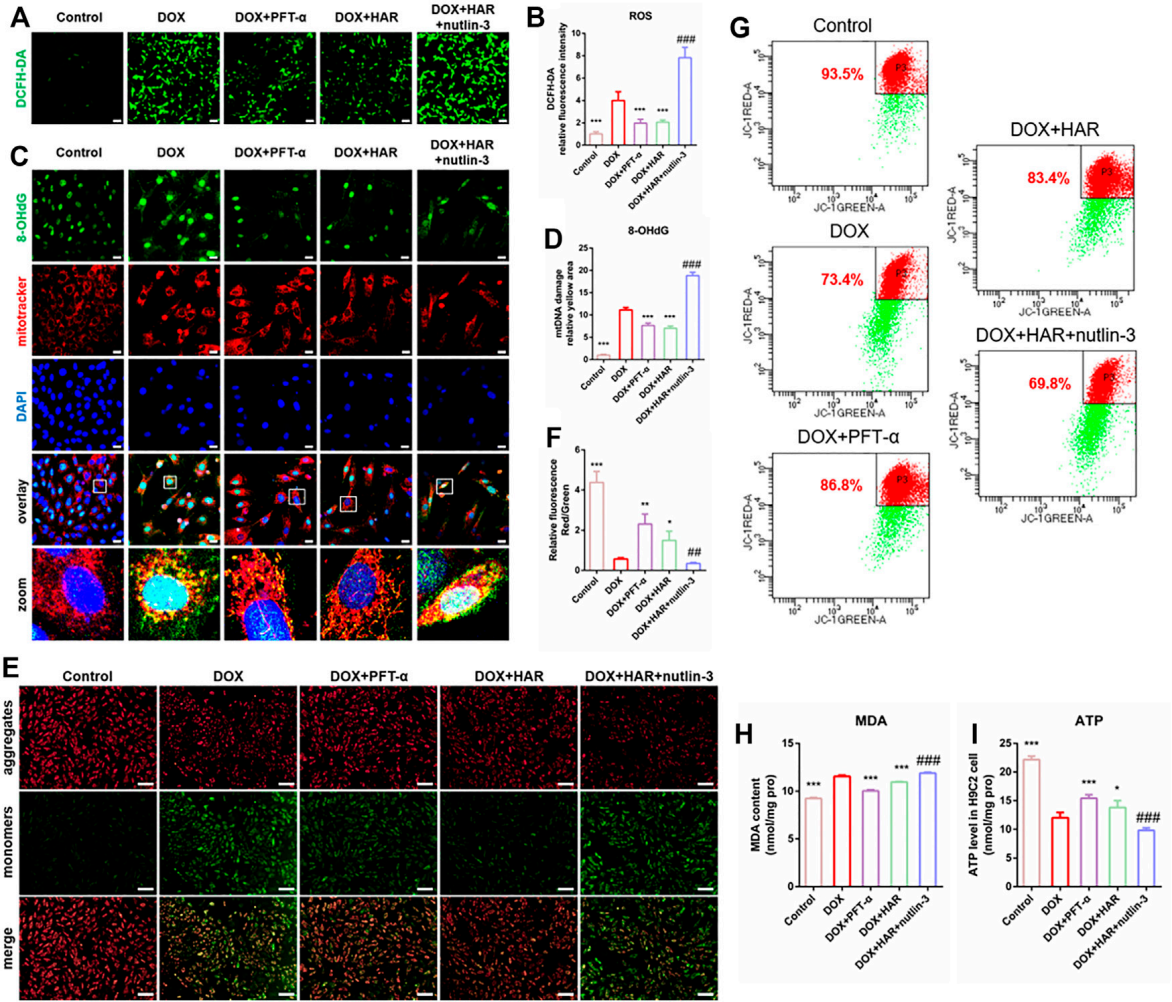
HAR maintained mitochondrial health by suppressing p53 *in vitro*. **(A–D)** Representative images and quantitative analysis of ROS in H9C2 cells measured by staining with DCFH-DA and mtDNA injury measured by costaining with 8-OhdG (DNA damage marker) and MitoTracker (*n* = 6). Scale bars: 100 and 25 μm. **(E–G)** Representative images and quantitative analysis of mitochondrial membrane potential in H9C2 cells measured by staining with JC-1 and detection under a fluorescence microscope and fluorescence-activated cell sorting (*n* = 4–6). Scale bar: 100 μm. **(H)** MDA was quantified in H9C2 cells to evaluate the degree of damage to intracellular membrane structures (*n* = 6). **(I)** The intracellular ATP content in various groups of H9C2 cells. ATP content reflects mitochondrial productivity. *p < 0.05, **p < 0.01, ***p < 0.001 vs. DOX group (*n* = 3–6). ^##^ *p* < 0.01, ^###^ *p* < 0.001 vs. (DOX + HAR) group. The data are shown as the mean ± s.d.

To investigate the mechanisms of the impact of DOX and HAR on apoptosis, we assessed the parameters of mitochondrial homeostasis. According to the data of Figure 5, ROS, MDA and damaged mtDNA (colocalization of 8-OhdG and MitoTracker) were increased in the DOX group compared to those in the control group. In contrast, ROS, MDA and damaged mtDNA were decreased in the HAR and PFT-α groups compared to those in the DOX group. Nutlin-3 reversed the effect of HAR (Figures 5A–D,H). Moreover, the levels of ATP and mitochondrial membrane potential, which was presented as the ratio of red to green fluorescence intensity by fluorescence microscopy or the percentage of red fluorescence by flow cytometry, were reduced in the DOX group compared with those in the control group. Conversely, ATP and mitochondrial membrane potential in the DOX + HAR and DOX + PFT-α groups were increased compared with those in the DOX group, and these changes were reversed by nutlin-3 (Figures 5E–G,I).

Overall, HAR inhibited the expression of p53 and alleviated DOX-induced damage to mitochondria, and these effects were reversed by nutlin-3. These results demonstrated that HAR reduced apoptosis induced by the detrimental impact of DOX on mitochondrial homeostasis, and the effect was associated with the suppression of p53 by HAR.

### HAR Alleviated DOX-Induced Apoptosis and Mitochondrial Oxidative Stress Damage by Promoting Parkin-Mediated Mitophagy *In Vitro*

**FIGURE 6:**
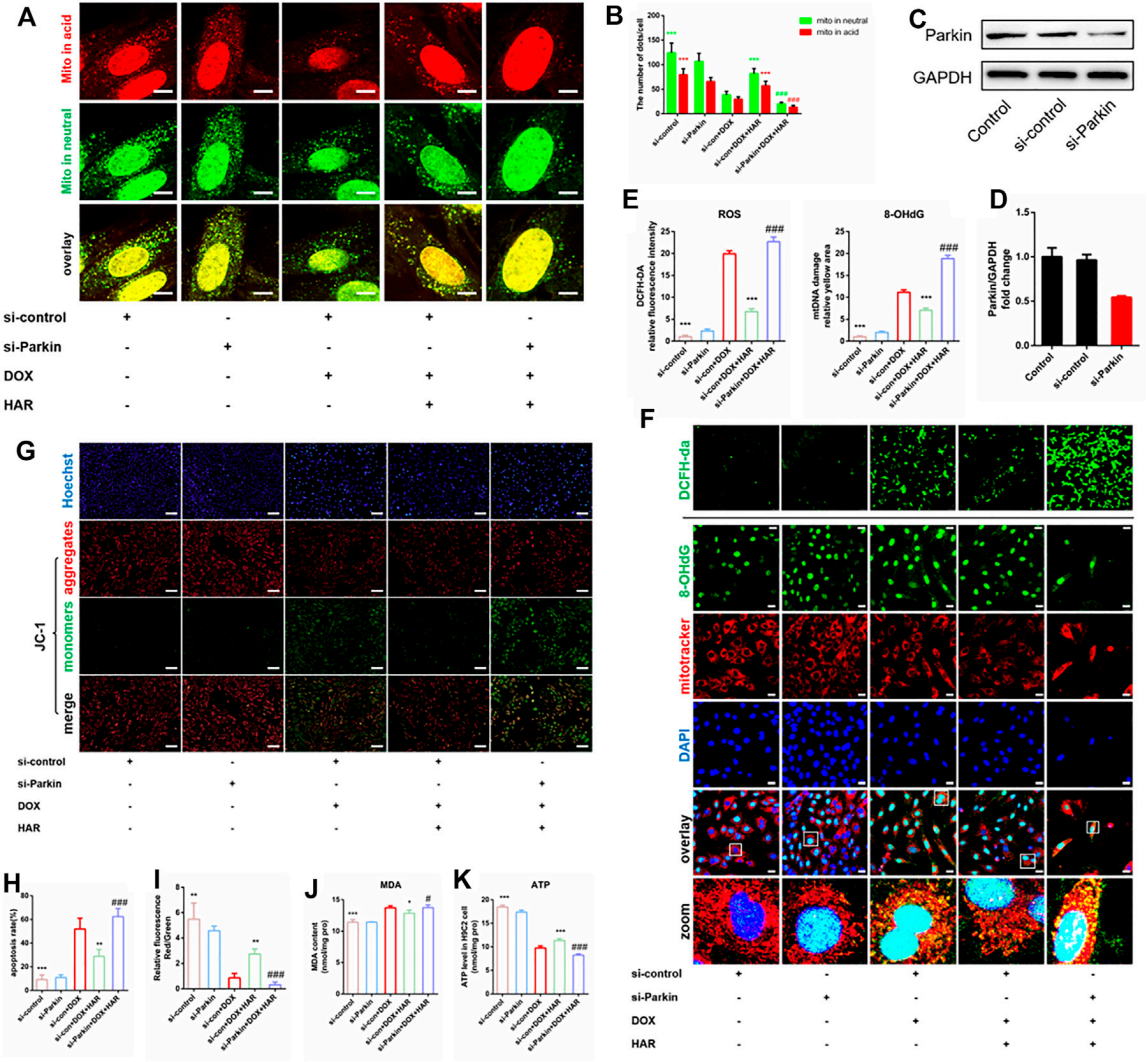
HAR alleviated DOX-induced apoptosis and mitochondrial oxidative stress damage by promoting Parkin-mediated mitophagy *in vitro*. **(A,B)** Representative confocal images and quantitative analysis of H9C2 cells infected with mt-Keima adenovirus before transfection with Parkin siRNA (*n* = 6). **(C,D)** Representative immunoblots and quantitative analysis of the protein level of Parkin in H9C2 cells treated with Parkin siRNA. **(E,F)** Representative confocal images and quantitative analysis of H9C2 cells stained with DCFH-DA and costaining of 8-OhdG and MitoTracker after infection with Parkin siRNA. Scale bars: 100 and 25 μM. **(G–I)** Representative images and quantitative analysis of H9C2 cells stained with JC-1 and Hoechst 33,342 after infection with Parkin siRNA (*n* = 6). Scale bar: 100 μm. **(J,K)** The intracellular contents of MDA and ATP in H9C2 cells (*n* = 6). *p < 0.05, **p < 0.01, ***p < 0.001 vs (si-con + DOX) group. ^#^ *p* < 0.05, ^###^ *p* < 0.001 vs. (si-con + DOX + HAR) group. The data are shown as the mean ± s.d.

To test whether HAR enhanced Parkin-mediated mitophagy is required for protective effects of HAR on mitochondria and apoptosis, we silenced Parkin using an siRNA in H9C2 cells. The protein level of Parkin was significantly reduced by si-Parkin (Figures 6C,D). The green puncta of Mt-Keima represent the sum of mitochondria not involved in mitophagy andmitophagosomes, while red puncta represent the mitophagosomes incorporated by the autolysosome (referred to as mitophagolysosomes during mitophagy) (Klionsky et al., 2021). As shown in Figures 6A,B, the promotion of mitophagolysosomes formation by HAR was apparently reversed by si-Parkin. We then assessed mitochondrial homeostasis by detecting alterations in cellular ROS, MDA, damaged mtDNA, mitochondrial membrane potential and ATP. As expected, a decrease in ROS, MDA and damaged mtDNA and an increase in the mitochondrial membrane potential and ATP were detected in the DOX + HAR group compared with those in the DOX group. However, si-Parkin treatment abrogated these protective effects of HAR on the mitochondria (Figures 6E–K). Furthermore, in agreement with these results, apoptosis induced by DOX was alleviated by HAR treatment, and this effect was reversed by si-Parkin treatment (Figures 6G,H). Consequently, we suggest that protective effects of HAR on mitochondrial homeostasis and alleviation of apoptosis were mediated by enhanced Parkin-mediated mitophagy.

### HAR Promoted Parkin-Mediated Mitophagy by Inhibiting the Binding of Cytosolic p53 to Parkin

**FIGURE 7:**
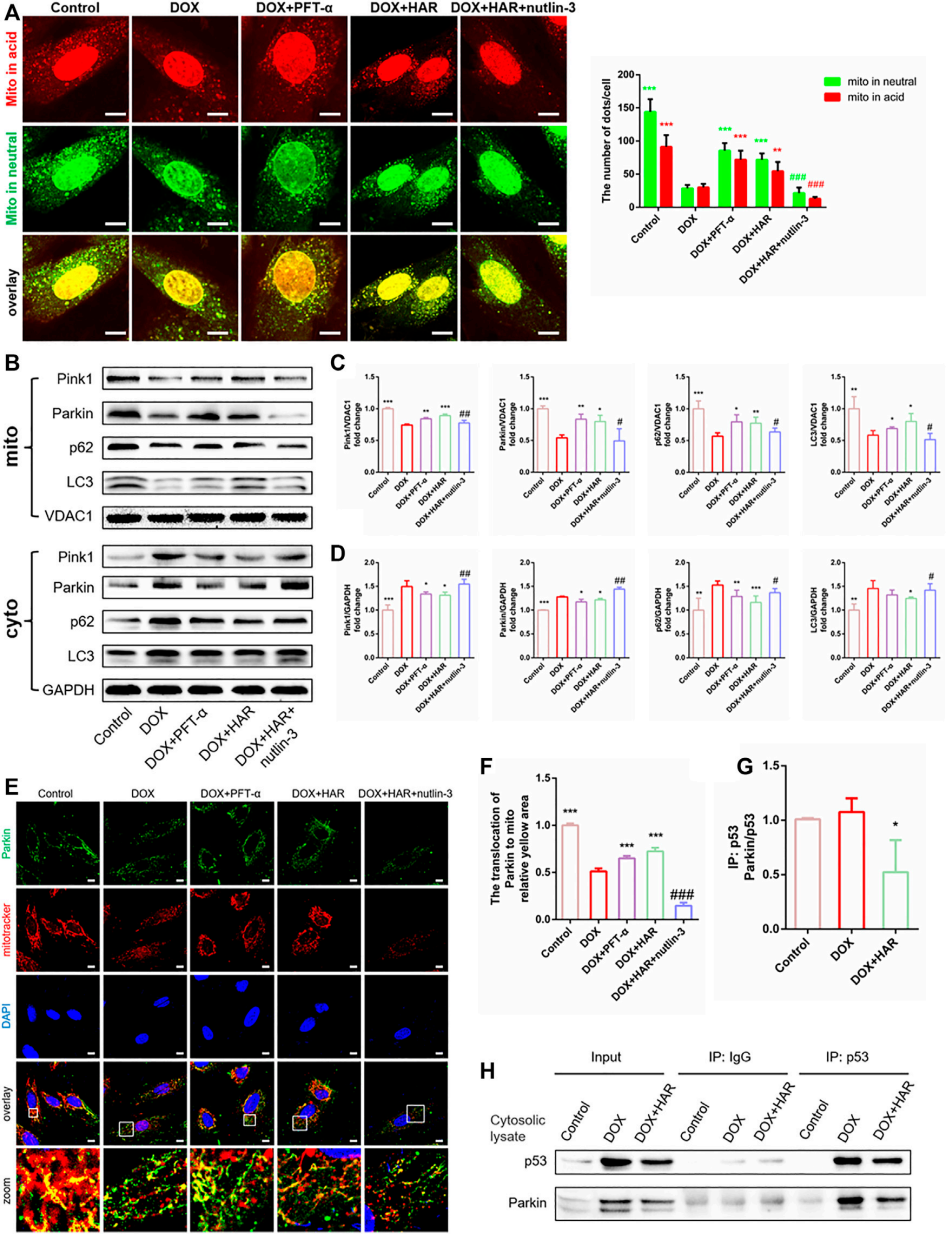
HAR promoted Parkin-mediated mitophagy by inhibiting the binding of cytosolic p53 to Parkin *in vitro*. **(A)** Representative confocal images and quantitative analysis of mt-Keima puncta expressed in H9C2 cells treated with HAR, PFT-α or nutlin-3 (*n* = 6). **(B–D)** p53-dependent mitophagy-related proteins located in the cytosol and mitochondria were determined by immunoblotting, and their relative protein expression levels are shown after normalization to GAPDH or VDAC1 (*n* = 3–6). **(E,F)** Quantitative analysis of Parkin recruited to the mitochondria by costaining for Parkin and MitoTracker (*n* = 6). Scale bar: 10 μm. **(G,H)** Representative immunoblots and quantitative analysis of the binding of p53 and Parkin in the cytosolic lysates of H9C2 cells. Cytosolic lysates of H9C2 cells were immunoprecipitated with anti-p53 and control IgG antibodies and immunoblotted with anti-Parkin and anti-p53 antibodies. The quantity of Parkin binding with p53 are normalizing to immunoprecipitated p53 (*n* = 3). *p < 0.05, **p < 0.01, ***p < 0.001 vs. DOX group. ^#^ *p* < 0.05, ^##^ *p* < 0.01, ^###^ *p* < 0.001 vs. (DOX + HAR) group. The data are shown as the mean ± s.d.

To test the relationship of Parkin and p53, we added PFT-α and nutlin-3 and examined the changes in Parkin-mediated mitophagy *in vitro*. As shown in mt-keima, mitophagosomes and mitophagolysosomes were markedly decreased in the DOX group compared with those in the control group. In contrast, they were significantly increased in the HAR and PFT-α groups compared with those in the DOX group, and nutlin-3 considerably decreased the number of mitophagosomes and mitophagolysosomes compared with those in the HAR treatment group (Figure 7A). Furthermore, we measured the levels of proteins involved in mitophagy, including Parkin, Pink1, LC3 and p62, in the mitochondria and cytosol (Figures 7B–D). Consistent with the data described above, the levels of Parkin, Pink1, LC3 and p62 were reduced in the mitochondria and were increased in the cytosol in the DOX group compared with those in the control group. However, an opposite result was observed in the HAR and PFT-α groups compared with that in the DOX group. Nutlin-3 reversed the effect of HAR. In addition, the results of colocalization of Parkin with mitochondria showed was decreased in the DOX group compared with that in the control group, confirming that DOX reduced the translocation of Parkin to the mitochondria. Similarly, HAR and PFT-α abolished the impact of DOX to some extent, and nutlin-3 decreased the effects of HAR (Figures 7E,F). These results indicated that the mitophagy-related proteins Parkin, Pink1, LC3 and p62 accumulated in the cytosol and reduced translocation to the mitochondria under DOX treatment, thus blocking the formation of mitophagosomesand mitophagolysosomes. However, HAR and PFT-α promoted the formation of mitophagosomes andmitophagolysosomes, and this effect was abrogated by nutlin-3.

A decrease of Parkin in whole cell and enhanced accumulation of Parkin in the cytosol implied that initiation of mitophagy may be blocked by inhibiting its transfer to the mitochondria. Furthermore, PFT-α played the same role as HAR, and activation of p53 by nutlin-3 reversed the effects of HAR. Then, we found that HAR promoted the translocation of Parkin to the mitochondria by inhibiting the binding of p53 to Parkin, thus restoring the mitophagy flux. In detail, we detect that the binding of p53 to Parkin in the DOX group was notably increased compared with that in the control group, and the binding of p53 to Parkin in the HAR group was decreased compared with that in the DOX group (Figure 7H). However, since the protein level of p53 is significantly altered upon DOX and HAR treatment, which implied that DOX and HAR may inhibit the binding of p53 and Parkin by interfering with p53 protein level. Then we normalized immunoprecipitated Parkin to corresponding p53, and found that relative ratio of Parkin to p53 between control and DOX group have no difference, while prominent decrease under HAR treatment compared to DOX group were observed (Figure 7G). The results indicated that the surge of p53 protein was entirely responsible for the increased binding of Parkin and p53 in DOX group, but differently in HAR group, not only decreased expression of p53 but probably directly disrupted binding of both proteins contribute to more prominently reduced combination of p53 and Parkin relative to decreased expression of p53. In summary, the results confirmed that HAR relieved the blockade of mitophagy by downregulating the expression of p53 as well as disrupted binding of p53 and Parkin in cytosol, thus enhancing the translocation of Parkin to the mitochondria.

### Protective Effect of HAR on DICT did Not Interfere With Antitumour Effect of DOX

**FIGURE 8:**
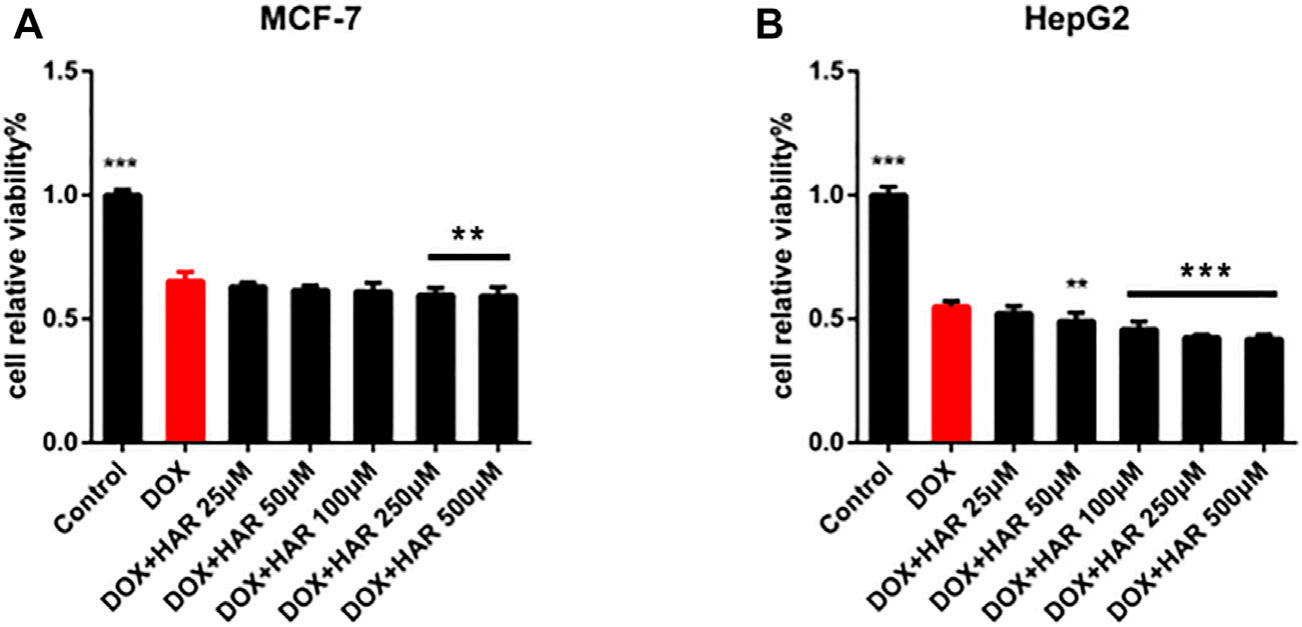
HAR played a protective role in the heart without interfering with antitumour effect of DOX. **(A,B)** The influence of HAR on the anticancer effect of DOX on MCF-7 and HepG2 cells was determined by CCK-8 assay (*n* = 6). *p < 0.05, **p < 0.01, ***p < 0.001 vs. DOX group. The data are shown as the mean ± s.d.

In addition to confirming the protective effect of HAR on cardiomyocytes, we evaluated whether HAR interferes with the anticancer effect of DOX on the human breast carcinoma cell line MCF-7 and human hepatocellular carcinoma cell line HepG2. As shown in Figure 8, HAR did not affect inhibition of cancer cell viability by DOX; moreover, HAR had a potential cooperative effect with DOX on inhibition of the growth of HepG2 and MCF-7 cancer cells (Figures 8A,B).

## Discussion and Conclusion

The application of DOX in clinical practice is severely limited due to its dose-dependent and progressive cardiotoxicity (Sorensen et al., 2003; Doyle et al., 2005). At present, dexrazoxane is the only FDA-approved drug for the prevention of DICT that reduces the formation of iron-anthracycline complexes, acting as an iron-chelating agent or interfering with topoisomerase 2β (Langer et al., 2000; Lyu et al., 2007; Getz et al., 2020). However, dexrazoxane has carcinogenic potential due to an increased risk for the development of acute myeloid leukaemia and myelodysplastic syndrome. This risk is accompanied by restrictions on the use of dexrazoxane by the FDA, and the American Society of Clinical Oncology recommends the drug only as a cardiac protective agent for patients receiving a high dose of DOX (Yu et al., 2020). Therefore, identification of additional cardioprotective agents for DICT has been of interest to oncologists and cardiologists. Our team discovered the impressive cardioprotective effect of HAR on DICT and confirmed that HAR mitigated DOX-induced mitochondrial damage through p53-Parkin-mediated mitophagy, providing evidence for future application of HAR in clinical trials.

Maintenance of a healthy and functional mitochondrial population is required to produce ATP to meet high energy demand of the heart. The majority of studies on DOX-induced cardiotoxicity have implicated ROS-mediated the formation of a vicious loop in which excessive ROS production can cause accumulation of mitochondrial superoxide, lipid peroxidation, increased damage of mtDNA, mitochondrial membrane potential depolarization and decreased ATP generation, resulting in increased ROS formation and apoptosis (Angsutararux et al., 2015; Gorini et al., 2018; Wallace et al., 2020). The regulation of mitophagy has become an effective strategy for the treatment of DICT. For example, liensinine, as an inhibitor of autophagy, markedly decreased the viability and increased apoptosis in breast cancer cells treated with various chemotherapeutic agents by increasing the accumulation of p62 and LC3 (Zhou et al., 2015). In our research, DOX (1 μM for 24 h), same as liensinine, promoted cardiomyocyte apoptosis in DOX-induced cardiotoxicity by inhibiting autophagy and then promoting the accumulation of LC3 and P62. While, liensinine has also been demonstrated significant cardioprotective effects in DOX (5 μM 24 h) -induced cardiotoxicity by inhibiting Drp1 mediated mitochondrial fission (Liang et al., 2020). These different results may be caused by DOX treatment in different kinds of cells and at different doses.

Parkin-mediated mitophagy is the core mechanism of mitochondrial quality control that targets impaired mitochondria and delivers them to the lysosomes for degradation to maintain mitochondrial homeostasis (Ashrafi and Schwarz, 2013; Bravo-San Pedro et al., 2017; Eid et al., 2019). Thereinto, mitochondrial translocation of Parkin is critical for mitophagy in many diseases (Hoshino et al., 2013; Eid et al., 2016). In DICT, activated cytosolic p53 has been shown to function as a blocker of mitochondrial translocation of Parkin, thus impairing the removal of compromised mitochondria (Hoshino et al., 2013). In addition, in most human tumours, the p53 gene is lost, mutated or inactivated; thus, various p53 species in the heart have become promising targets for the pharmacological treatment of cardiotoxicity caused by chemotherapeutic drugs without interfering with anticancer activity (Levine et al., 1994; Ferber, 1999). Our results confirmed that HAR did not interfere with inhibition of viability of cancer cells, such as MCF-7 and HepG2 cells, by DOX and even synergistically promoted the death of MCF-7 and HepG2 cells (Figure 8). Therefore, the present study focused on the mechanisms involved in DICT, including mitochondrial oxidative damage, disturbances of the mitophagy flux, and investigated whether HAR facilitates the clearance of unhealthy mitochondria injured by DOX through p53-Parkin-mediated mitophagy to ultimately reduce apoptosis.

Initially, we applied the DICT zebrafish model established previously to screen out HAR, which had better cardioprotective efficacy than DXZ, and further verified its significant cardioprotective effect and molecular mechanism in DICT cells and mouse model. A p53 inhibitor (PFT-α) and an agonist (nutlin-3) were used as positive and negative controls, respectively, and the results verified the protective effect of HAR on mitophagy, mitochondrial damage and apoptosis involves inhibition of p53. Then, siRNA of Parkin was applied, and the results demonstrated that restoration of DOX-induced alterations in mitochondrial homeostasis and apoptosis were associated with enhancement of Parkin-mediated mitophagy by HAR. Furthermore, HAR prominently diminished Parkin binding to p53 in the cytosol by not only decreasing expression of p53 but probably directly disrupting binding of both proteins. Therefore, we believed that HAR inhibits the signal cascade of mitophagy blocking-mitochondrial oxidative damage intensification-apoptosis activation by inhibiting the expression of p53 and directly interdicting the protein binding of p53 and Parkin.

The present study for the first time investigated HAR for the treatment of DICT and show that HAR has a protective effect on DICT cells, zebrafish and mice mediated by a novel molecular mechanism of protein-protein interactions. Specifically, HAR inhibits the binding of cytoplasmic p53 to Parkin, thus alleviating the blockade of mitophagy induced by DOX and promoting the degradation of damaged mitochondria to ultimately relieve cardiac dysfunction in DICT models.

## Data Availability

The original contributions presented in the study are included in the article/Supplementary Material, further inquiries can be directed to the corresponding authors.
