# Supplementary figures and images for: Harpagoside Protects Against Doxorubicin-Induced Cardiotoxicity via P53-Parkin-Mediated Mitophagy
Source: Front Cell Dev Biol. 2022 Feb 10;10:813370. doi: 10.3389/fcell.2022.813370 (PMC8867983; doi:10.3389/fcell.2022.813370)

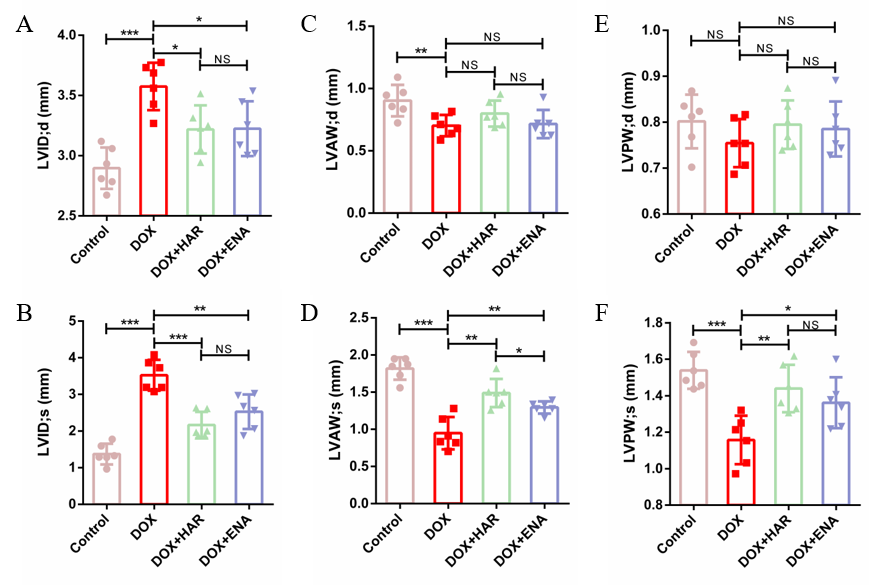

Supplement: Supplementary file 1 [file Image1.tif]
